# Hydroxychloroquine attenuates renal ischemia/reperfusion injury by inhibiting cathepsin mediated NLRP3 inflammasome activation

**DOI:** 10.1038/s41419-018-0378-3

**Published:** 2018-03-02

**Authors:** Tao-Tao Tang, Lin-Li Lv, Ming-Ming Pan, Yi Wen, Bin Wang, Zuo-Lin Li, Min Wu, Feng-Mei Wang, Steve D Crowley, Bi-Cheng Liu

**Affiliations:** 1 Institute of Nephrology, Zhong Da Hospital, Southeast University School of Medicine, Nanjing, China; 2 Division of Nephrology, Department of Medicine, Duke University and Durham VA Medical Centers, Durham, NC United States

## Abstract

Inflammation is a major contributor to the pathogenesis of ischemic acute kidney injury (AKI), which complicates the post-operative outcomes of large numbers of hospitalized surgical patients. Hydroxychloroquine (HCQ), a well-known anti-malarial drug, is commonly used in clinical practice for its anti-inflammatory actions. However, little is known about its role in renal ischemia/reperfusion (I/R) injury. In the current study, mice were subjected to I/R injury and HCQ was administered for seven days by gavage prior to surgery. In parallel, HK-2 human renal proximal tubule cells were prophylactically treated with HCQ and then were exposed to hypoxia/reoxygenation (H/R). The results showed that HCQ significantly attenuated renal dysfunction evidenced by blunted decreases in serum creatinine and kidney injury molecular-1 expression and the improvement of HK-2 cell viability. Additionally, HCQ markedly reduced macrophage and neutrophil infiltration, pro-inflammatory cytokine production, and NLRP3 inflammasome activation. Mechanistic studies showed that HCQ could inhibit the priming of the NLRP3 inflammasome by down-regulating I/R or H/R-induced NF-κB signaling. Moreover, HCQ reduced cathepsin (CTS) B, CTSD and CTSL activity, and their redistribution from lysosomes to cytoplasm. CTSB and CTSL (not CTSD) were implicated in I/R triggered NLRP3 inflammasome activation. Notably, we found that HCQ attenuated renal injury through downregulation of CTSB and CTSL-mediated NLRP3 inflammasome activation. This study provides new insights into the anti-inflammatory effect of HCQ in the treatment of AKI.

## Introduction

Renal ischemia/reperfusion (I/R) injury, the major cause of acute kidney injury (AKI), is associated with severe morbidity and mortality in both developing and developed countries^1^. Accumulating evidence has suggested that inflammation plays a critical role in the pathology of ischemic injury^2–4^. However, effective therapies that improve AKI outcomes by attenuation of inflammation are still limited.

Chloroquine (CQ) and its analog hydroxychloroquine (HCQ), the anti-malarial drugs, were shown to have various anti-inflammatory and immunomodulatory effects, and currently have been widely used in the treatment of rheumatoid arthritis and systemic lupus erythematosus^5,6^. According to previous studies, actions of HCQ on the immune system appear to involve their ability to interfere with lysosomal acidification and inhibition of antigen presentation^7,8^, down-regulation of cytokine production and secretion by monocytes and T cells^9,10^, and inhibition of toll-like receptors signaling^11^. In addition, CQ and HCQ were shown to have potential beneficial effects in I/R injury of different organs^12–14^. Fang et al. reported that CQ treatment could ameliorate liver I/R injury by reducing inflammatory cytokine production^13^. However, the potential effect of these drugs on renal inflammation and injury remains largely unknown.

The NACHT, LRR, and PYD domains-containing protein 3 (NLRP3) inflammasome, is a cytoplasmic macromolecular complex that orchestrates early inflammatory responses of the innate immune system by inducing caspase-1 activation and IL-1β maturation^15–17^. Various danger signals, including mitochondrial reactive oxygen species (ROS)^18^, potassium efflux^19^, and the release of lysosomal cathepsins^20^, are identified as possible activators of the NLRP3 inflammasome. Notably, the important role of the NLRP3 inflammasome in modulating kidney inflammation has been confirmed in different renal disease models including I/R injury^21–27^. Iyer et al.^25^. demonstrated that necrotic tubular cells were capable of activating NLRP3 inflammasome in macrophages through the release of viable mitochondria. NLRP3-deficiency protected mice against renal inflammation and tissue damage after I/R injury^25,26^. Moreover, Bakker et al.^27^ reported that NLRP3 showed a tissue-specific role in which leukocyte-associated NLRP3 was responsible for tubular apoptosis, whereas renal-associated NLRP3 impaired wound healing. The absence of NLRP3 in tubular cells improved regenerative response^27^. These findings suggest that NLRP3 inflammasome could be a potential target for the treatment of renal I/R injury.

In this study, we explored the potential effects and the underlying mechanism of HCQ on renal inflammation in ischemic AKI. Our findings demonstrated that HCQ attenuates renal I/R injury by inhibiting cathepsin-mediated NLRP3 inflammasome activation, which provides a novel insight in understanding the anti-inflammatory effect of HCQ in AKI.

## Results

### HCQ protects I/R-induced acute kidney injury

**Fig. 1:**
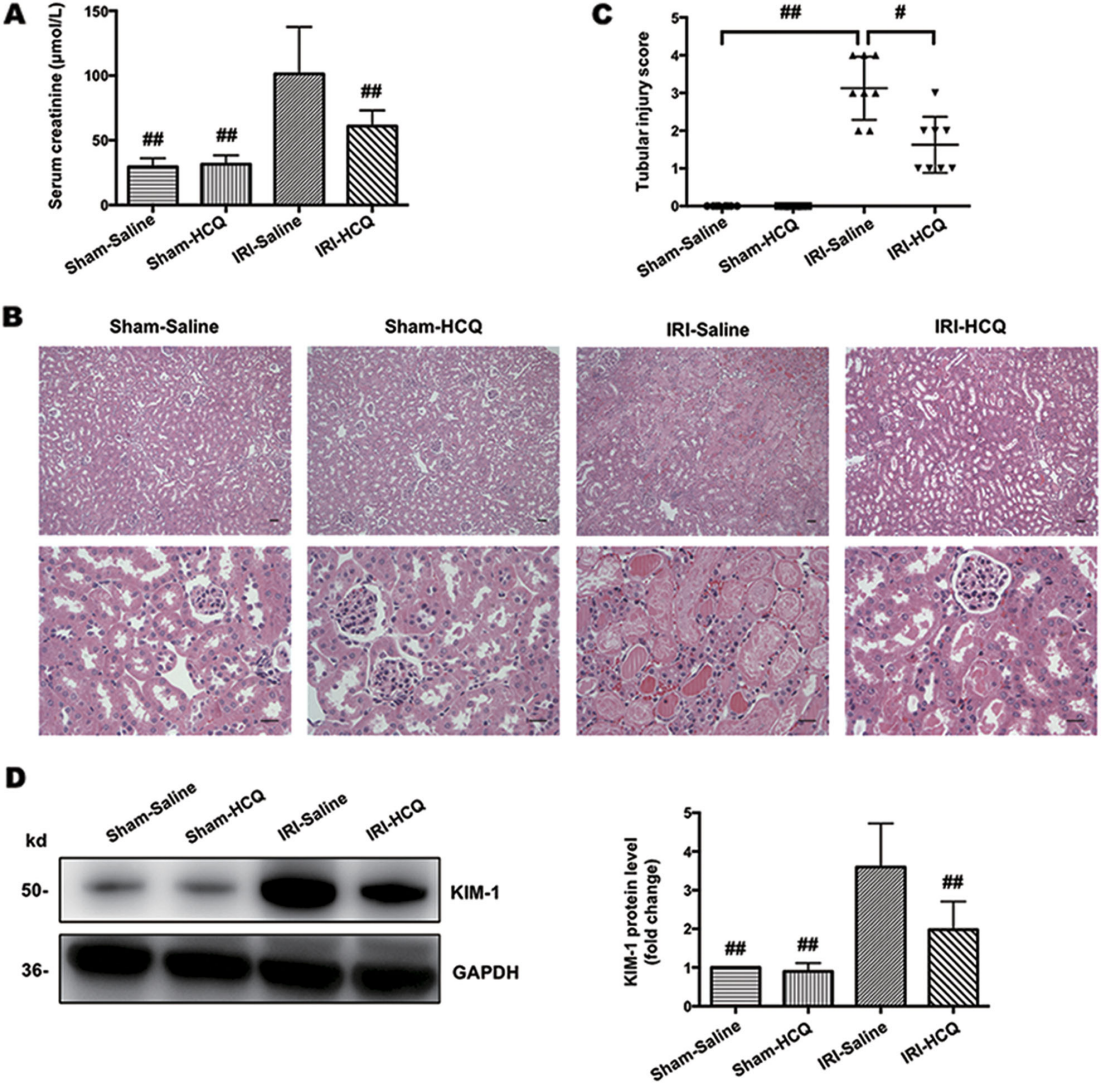
HCQ ameliorates renal I/R injury. **a** Effects of HCQ on serum creatinine after renal I/R injury. **b** H&E staining of the kidney. (Upper panel: ×100, Bars = 200 μm; Lower panel: ×400, Bars = 100 μm). **c** Quantification of tubular injury. Data are presented as the mean ± SD (*n* = 8). ^#^*p* < 0.05, ^##^*p* < 0.01 compared with the IRI-Saline group. **d** Western blots of KIM-1 in renal lysates. Data are presented as the mean ± SD (*n* = 5). ^##^*p* < 0.01 compared with the IRI-Saline group

As shown in Fig. 1a, serum creatinine was significantly increased in the IRI-Saline group, an effect that was attenuated in the HCQ-pretreated group. The kidney histopathological changes included necrosis and detachment of TECs, disappearance of the brush border, cellular debris accumulation and protein cast formation in the IRI-Saline group. These changes were dramatically limited by HCQ pretreatment (Fig. 1b, c). In addition, following I/R injury, the induction of kidney injury molecular-1 (KIM-1), a biomarker of proximal tubular injury, was also significantly blunted with HCQ therapy (Fig. 1d).

### HCQ attenuates I/R-induced renal inflammation

**Fig. 2:**
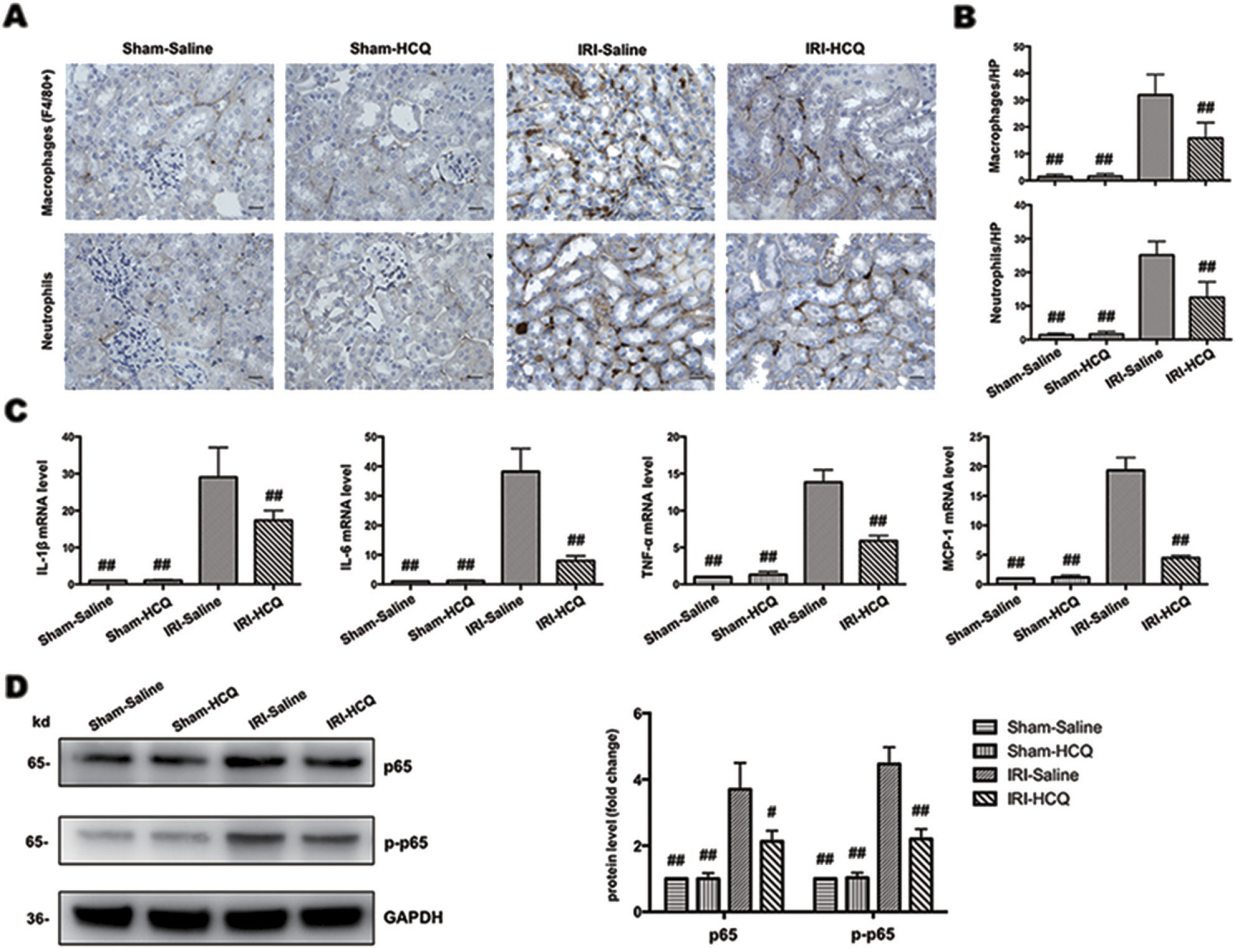
HCQ attenuates inflammation in experimental mice. **a** Immunohistochemistry staining of macrophages and neutrophils in renal interstitium (Original image magnification: × 400, Bars = 100 μm). **b** The number of macrophages and neutrophils in the four groups. **c** Effects of HCQ on the mRNA levels of IL-1β, IL-6, TNF-α, and MCP-1 in vivo. **d** Western blots of NF-κB p65 and p-p65 in renal lysates. Data are presented as the mean ± SD (*n* = 5). ^#^*p* < 0.05, ^##^*p* < 0.01 compared with IRI-Saline group

Immunostaining study in kidney sections demonstrated that the interstitial infiltration of inflammatory cells, such as macrophages (F4/80 positive) and neutrophils, following I/R injury were significantly diminished by HCQ pretreatment (Fig. 2a,b) as were mRNA levels of IL-1β, IL-6, TNF-α, and MCP-1 (Fig. 2c). As IL-6, TNF-α, and MCP-1 are markers of NF-κB signaling, we further detected NF-κB p65 and p-p65 protein expression. It was shown that the expression of p65 and phosphorylation of p65 were substantially decreased in IRI-HCQ group compared with IRI-Saline group (Fig. 2d).

### HCQ protects HK-2 cells from H/R injury and regulates NF-κB signaling

**Fig. 3:**
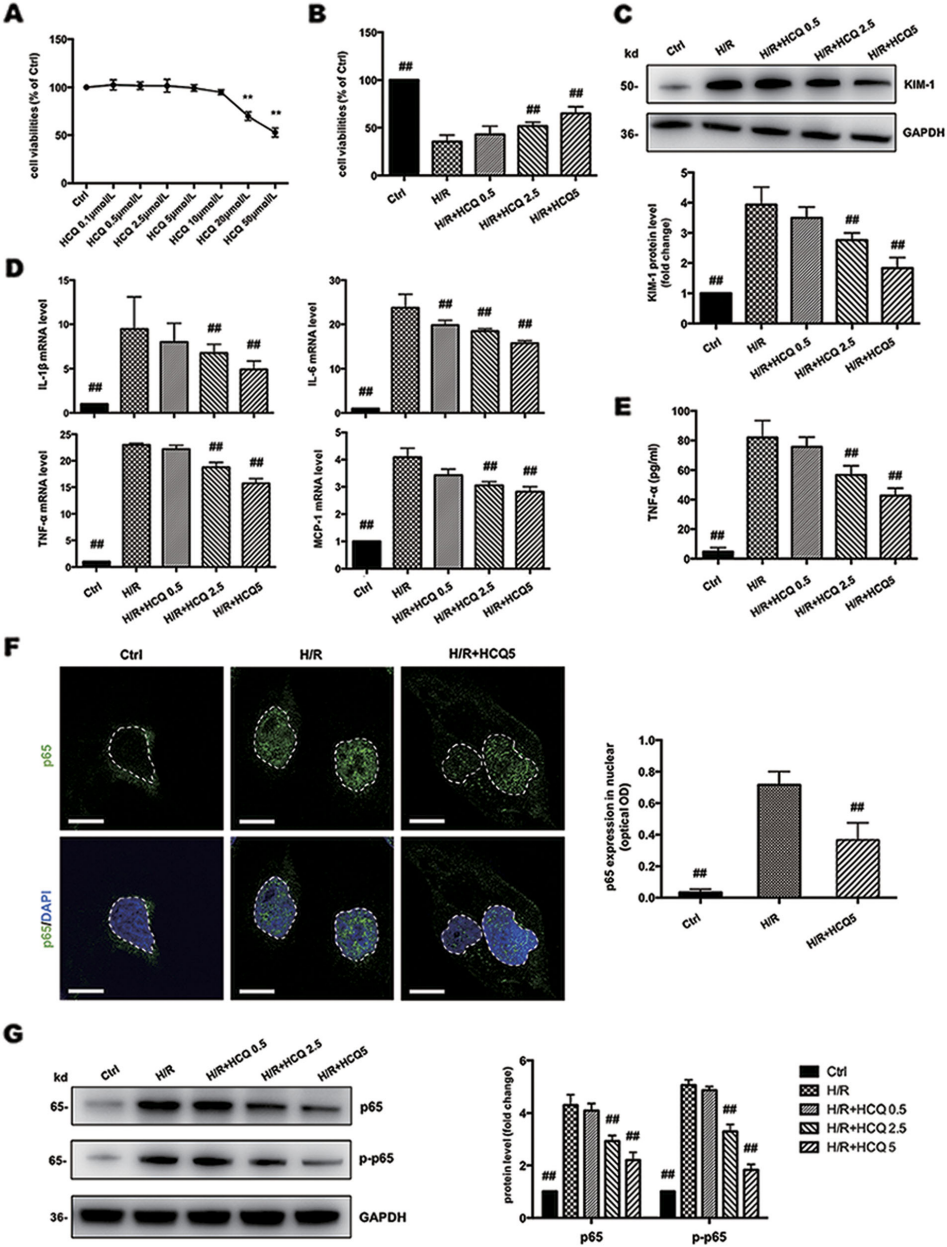
HCQ protects HK-2 cells from H/R injury and regulates NF-κB signaling. **a** Effects of HCQ on the cell viabilities of HK-2 cells. Data are presented as the mean ± SD (*n* = 3). **p < 0.01 compared with control group. **b** Effects of HCQ on the cell viabilities of HK-2 cells against H/R injury. **c** Western blots of KIM-1 in cell lysates. **d** Effects of HCQ on the mRNA levels of IL-1β, IL-6, TNF-α, and MCP-1 in HK-2 cells. **e** ELISA detection of TNF-α expression in supernatants. **f** Effects of HCQ on the nuclear translocation of NF-κB p65 in HK-2 cells based on confocal microscopy. White broken lines indicate borders of the nucleus. The optical density of p65 expression in the nuclear was quantified. (Bars = 30 μm) **g** Western blots of NF-κB p65 and p-p65 in cell lysates from HK-2 cells stimulated with H/R and pretreated with HCQ. Data are presented as the mean ± SD (*n* = 3).^##^ *p* < 0.01 compared with H/R group

First, a CCK-8 assay was used to assess the cytotoxicity of HCQ. At concentrations from 0.1 to 10 μmol/L, HCQ showed no obvious cytotoxicity on HK-2 cells (Fig. 3a). Hypoxia/reoxygenation (H/R) injury significantly decreased the cell viability compared to control cells. Pretreatment with HCQ improved cell viability in a dose-dependent manner (Fig. 3b). Therefore, HCQ concentrations at 0.5, 2.5, and 5 μmol/L were selected to protect HK-2 cells against H/R injury. Additionally, KIM-1 expression was significantly decreased by HCQ at the concentration of 2.5 and 5 μmol/L (Fig. 3c).

In addition, we detected expression levels of IL-1β, IL-6, TNF-α, and MCP-1, which were decreased by HCQ pretreatment in a dose-dependent manner (Fig. 3d). We also found that HCQ pretreatment dose dependently reduced TNF-α in culture supernatants (Fig. 3e). The protein expression of NF-κB p65 and p-p65 and the nuclear translocation of NF-κB p65 were substantially repressed in HCQ-pretreated groups (Fig. 3f, g).

### HCQ inhibits NLRP3 inflammasome activation in vivo and in vitro

**Fig. 4:**
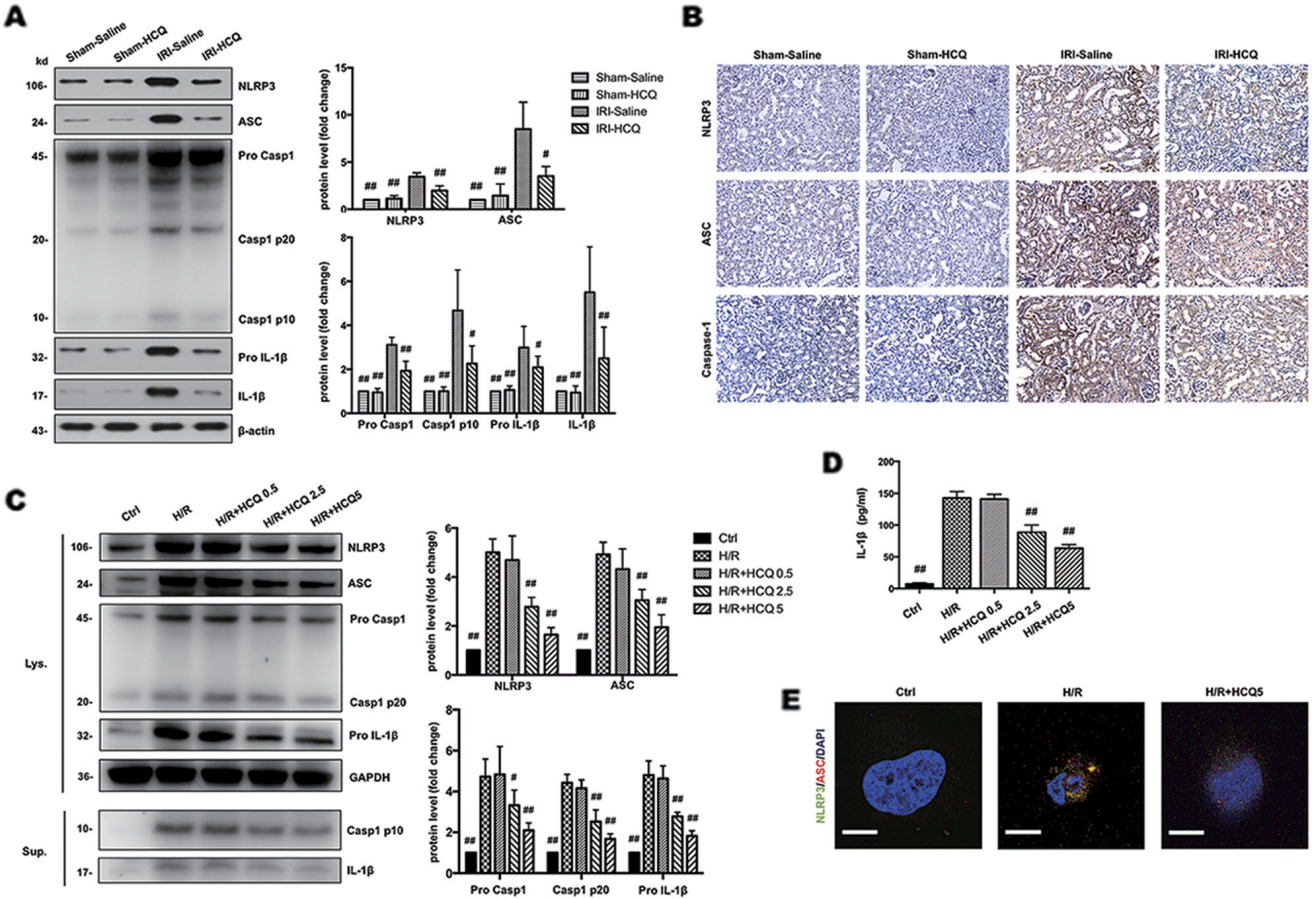
HCQ inhibits NLRP3 inflammasome activation in vivo and in vitro. **a** Western blots of NLRP3, ASC, Caspase-1 and IL-1β in renal lysates. Data are presented as the mean ± SD (*n* = 5). ^#^*p* < 0.05, ^##^*p* < 0.01 compared with IRI-Saline group. **b** Immunohistochemistry analysis of NLRP3, ASC and Caspase-1 expression in kidney tissue (Original image magnification: × 200, Bars = 100 μm). **c** Western blots of cell lysates (Lys.) and supernatants (Sup.) from HK-2 cells stimulated with H/R and pretreated with HCQ. **d** ELISA detection of IL-1β expression in supernatants. **e** Effects of HCQ on NLPR3 and ASC expression in HK-2 cells were observed by confocal microscopy (Bars = 30 μm). Data are presented as the mean ± SD (*n* = 5). ^#^*p* < 0.05, ^##^*p* < 0.01 compared with H/R group

We measured the expression of NLRP3, ASC, caspase-1, and IL-1β in the kidney lysates and found that they were noticeably elevated in the IRI-Saline group and were significantly suppressed by HCQ pretreatment (Fig. 4a). Immunohistochemistry staining showed that NLRP3, ASC and caspase-1 were downregulated in tubules of IRI-HCQ group compared with that in IRI-Saline group (Fig. 4b). To demonstrate the effect of HCQ on NLRP3 inflammasome activation, we performed in vitro studies by treating HK-2 cells with different doses of HCQ before H/R performed. It was shown that the protein expression of NLRP3, ASC, pro-caspase-1, pro-IL-1β in cell lysates and caspase-1 p10, IL-1β in the supernatants were downregulated by HCQ in a dose-dependent manner (Fig. 4c). Moreover, production of IL-1β in culture supernatants (as measured by ELISA) was also dose dependently reduced by HCQ pretreatment (Fig. 4d), indicating NLRP3 inflammasome activation was inhibited. Whereas protein expression of NLRP3 inflammasome components and mRNA levels of pro-inflammatory cytokines was not substantially affected by HCQ treatment under normal condition. (Supplementary Fig. 1). In addition, immunofluorescence showed that NLRP3 expression and ASC speck formation were co-localized in the perinuclear space after H/R injury, whereas these changes were attenuated by HCQ pretreatment (Fig. 4e).

### CTSB and CTSL are involved in H/R triggered NLRP3 inflammasome activation

**Fig. 5:**
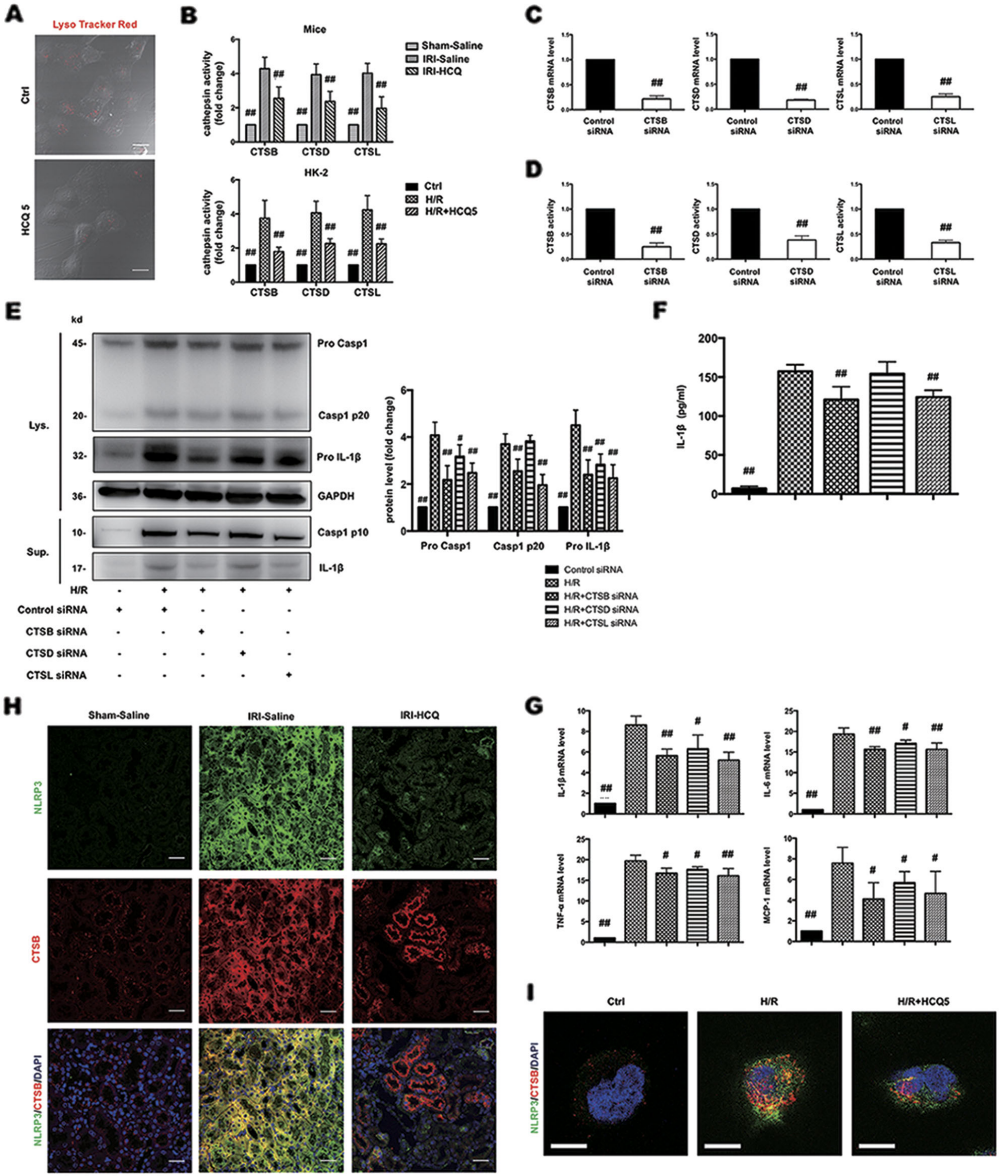
CTSB and CTSL are involved in H/R triggered NLRP3 inflammasome activation. **a** HK-2 cells were treated with HCQ for 12 h. Incubation with LysoTracker Red for 1 h and the intensity of cell fluorescence was observed (Bars = 50 μm). **b** Effects of HCQ on CTSB, D and L activity in experimental mice kidney and HK-2 cells. Data are presented as the mean ± SD (*n* = 8). ^##^*p* < 0.01 compared with the IRI-Saline and H/R group. **c**, **d** mRNA levels and activity of each cathepsin after siRNA transfection. Data are presented as the mean ± SD (*n* = 3). ## p < 0.01 compared with the control siRNA group. **e** Western blots of cell lysates (Lys.) and supernatants (Sup.) from HK-2 transfected with siRNA and stimulated with H/R. **f** ELISA detection of IL-1β levels in supernatants. **g** mRNA levels of IL-1β, IL-6, TNF-α, and MCP-1 in siRNA-transfected HK-2 cells and stimulated with H/R. Data are presented as the mean ± SD (*n* = 5). ^#^*p* < 0.05, ^##^*p* < 0.01 compared with the control siRNA-H/R group. **h, i** Effects of HCQ on the co-localization of NLRP3 and CTSB in vivo (Original image magnification: ×400, Bars = 100 μm) and in vitro (Bars = 30 μm)

Next we sought to determine the mechanism through which HCQ inhibits NLRP3 inflammasome activation. Various danger signals, including mitochondrial ROS, potassium efflux, and the release of lysosomal cathepsins (CTS), were identified as possible activators of the NLRP3 inflammasome^28^. As a weak base, HCQ could interfere with lysosomal acidification and reduce CTS activity^6^. Thus, we hypothesized that HCQ inhibits NLRP3 inflammasome activation by downregulation of CTS activity. However, which cathepsin triggers the activation of NLRP3 inflammasome in renal I/R injury is not clear. First, we tested CTSB, CTSD, and CTSL that have been linked to I/R injury according to previous studies^29–32^. We used LysoTracker Red to label acidic intracellular compartments (lysosomes) in HK-2 cells. Punctated red fluorescence marking lysosomes was clearly reduced under HCQ (5 μmol/L) treatment (Fig. 5a). In addition, the activity of CTSB, CTSD, and CTSL in whole kidney and in HK-2 cells were significantly decreased by HCQ pretreatment (Fig. 5b).

We transfected CTSB, CTSD, and CTSL siRNA into HK-2 cells to determine which one was responsible for H/R-induced NLRP3 inflammasome activation. siRNA transfection reduced the mRNA levels and the activity of each CTS (Figs. 5c, d). Expression of cleaved caspase-1 and IL-1β in the supernatants were significantly inhibited by CTSB and CTSL deficiency (Figs. 5e, f). However, protein expression of pro-caspase-1 and pro-IL-1β and mRNA expression of IL-1β, IL-6, TNF-α, and MCP-1 was all downregulated by three cathepsin siRNA transfection (Figs. 5e, g). These observations indicated that CTSB and CTSL are capable of inducing NLRP3 inflammasome activation. Although CTSD is not an activator of NLRP3 inflammasome, its absence could also attenuate inflammatory responses under H/R stimulation. Whereas inhibition of each CTS showed no significant effects on expression of NLRP3 inflammasome components under normal condition. (Supplementary Fig. 2). Furthermore, we observed the expression and location of NLRP3 and CTSB in kidney tissues and HK-2 cells. In control groups, CTSB immunoreactivity displayed a punctate pattern and very little association with NLRP3. In contrast, CTSB exerted a diffuse immunostaining pattern after I/R or H/R injury, suggesting a release from the lysosome to the cytosol, leading to co-localization with NLRP3. These changes were obviously abrogated by HCQ pretreatment (Figs. 5h, i).

### Inhibition of CTSB and CTSL attenuates renal I/R injury and blocks NLRP3 inflammasome activation

**Fig. 6:**
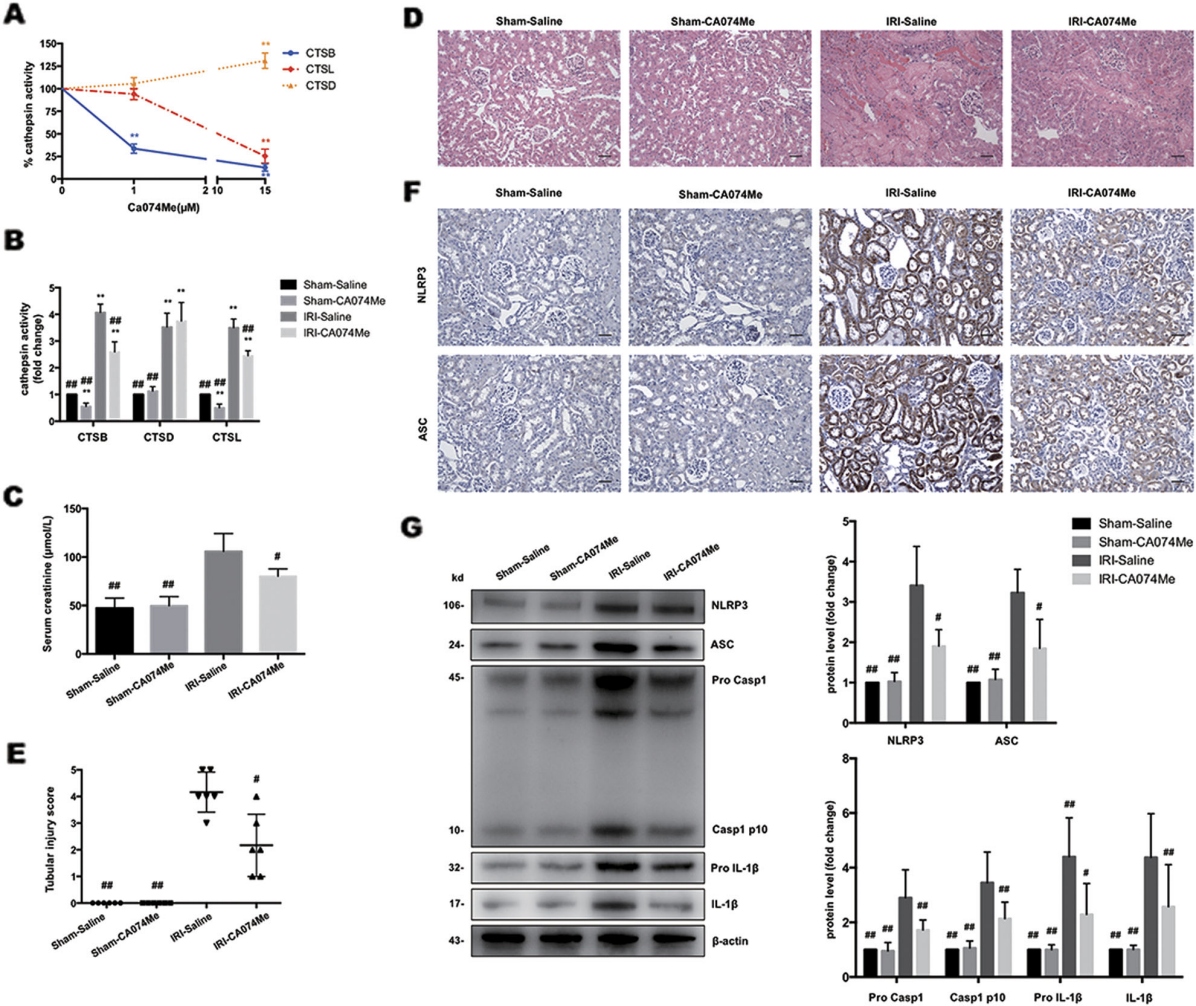
Inhibition of CTSB and CTSL attenuates renal I/R injury and blocks NLRP3 inflammasome activation. **a** Effects of different doses of Ca074Me on cathepsin (**b**, **d**, **l**) activity in HK-2 cells. Data are presented as the mean ± SD (*n* = 3). **p < 0.01 compared with the control group. **b** Effects of Ca074Me on cathepsin (**b**, **d**, **l**) activity in mice kidney. Data are presented as the mean ± SD (*n* = 6). **p < 0.01 compared with the Sham-Saline group, ^##^*p* < 0.01 compared with the IRI-Saline group. **c** Effects of HCQ on serum creatinine after renal I/R injury. **d** H&E staining of the kidney (Original image magnification: ×200, Bars = 200 μm). **e** Quantification of tubular injury. Data are presented as the mean ± SD (*n* = 6). ^#^*p* < 0.05, ^##^*p* < 0.01 compared with the IRI-Saline group. **f** Immunohistochemistry analysis of NLRP3 and ASC expression in kidney tissue (Original image magnification: × 200, Bars = 200 μm). **g** Western blots of NLRP3, ASC, Caspase-1, and IL-1β in renal lysates. Data are presented as the mean ± SD (*n* = 3). ^#^*p* < 0.05, ^##^*p* < 0.01 compared with IRI-Saline group

To address whether inhibition of CTSB and CTSL could block NLRP3 inflammasome activation in vivo, we pretreated mice with a cathepsin inhibitor. Ca074Me is recognized as a CTSB-specific inhibitor and has been used to implicate CTSB in NLRP3 activation in research. However, emerging evidence demonstrated that Ca074Me inhibits multiple cathepsins at high doses^33,34^. We found that CTSB activity in HK-2 cells was selectively inhibited by Ca074Me at low concentrations (<1 μmol/L), and at higher concentrations it inhibited CTSL activity as well (Fig. 6a). Consistently, CTSB and CTSL activity in kidney were down-regulated by Ca074Me pretreatment (Fig. 6b). As shown in Fig. 6c–e, serum creatinine and histopathological changes were substantially attenuated with Ca074Me pretreatment. Moreover, expression of NLRP3, ASC, caspase-1 and IL-1β in kidney as measured by immunohistochemistry staining (Fig. 6f) and western blotting (Fig. 6g) were noticeably suppressed by Ca074Me pretreatment. These data demonstrated that inhibition of CTSB and CTSL effectively blocked I/R injury triggered NLRP3 inflammasome activation.

### HCQ inhibits H/R-induced NLRP3 inflammasome activation by down-regulation of CTSB and CTSL activity

**Fig. 7:**
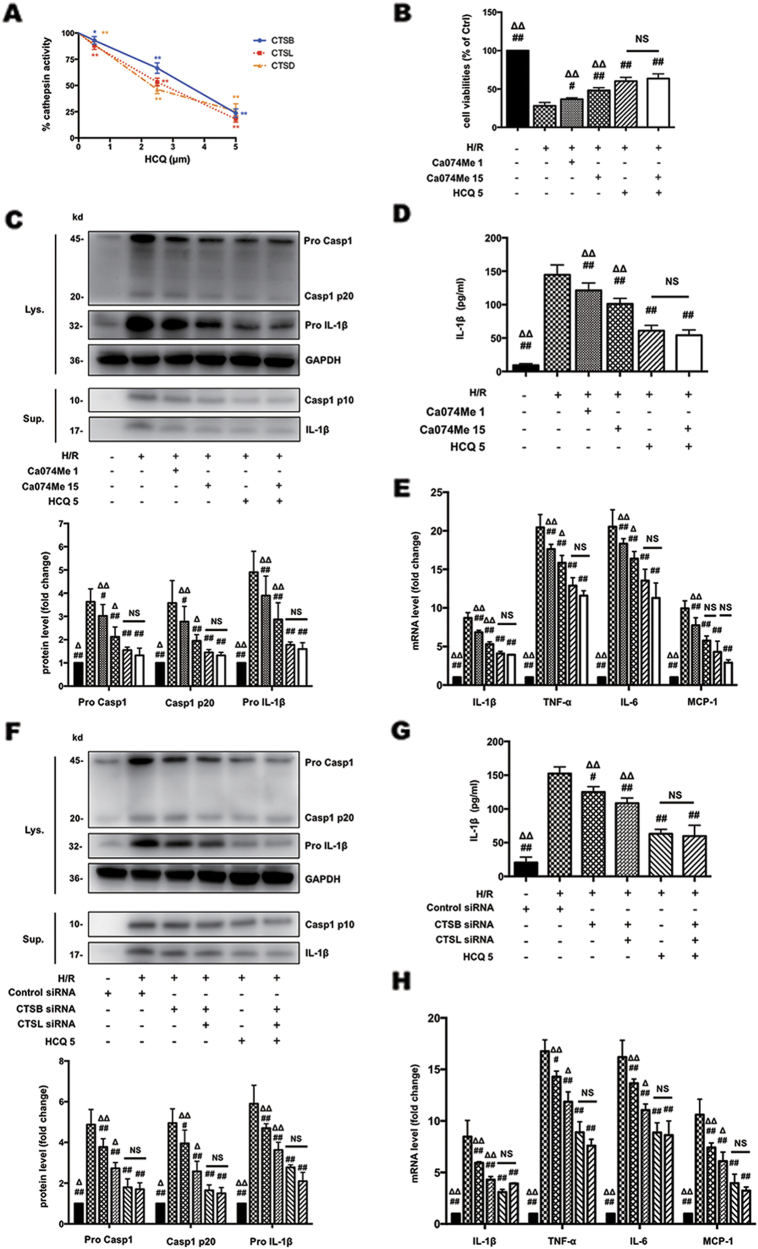
HCQ inhibits NLRP3 inflammasome activation by down-regulation of CTSB and CTSL activity. **a** Effects of HCQ on cathepsin (**b**, **d**, **l**) activity in HK-2 cells at different doses. Data are presented as the mean ± SD (*n* = 5). *p < 0.05, **p < 0.01 compared with the control group. **b** Cell viabilities of HK-2 cells stimulated with H/R and pretreated with HCQ and/or Ca074Me. **c** Western blots of cell lysates (Lys.) and supernatants (Sup.) from HK-2 cells stimulated with H/R and pretreated with HCQ and/or Ca074Me. **d** ELISA detection of IL-1β levels in supernatants. **e** Effects of HCQ on the mRNA levels of IL-1β, IL-6, TNF-α, and MCP-1 with or without Ca074Me. **f** Western blots of cell lysates (Lys.) and supernatants (Sup.) from HK-2 cells pretreated with HCQ and/or transfected with control siRNA or siRNA targeting CTSB or CTSL. **g** IL-1β levels in the supernatants as measured by ELISA. **h** Effects of HCQ on the mRNA levels of IL-1β, IL-6, TNF-α, and MCP-1 with or without siRNA transfection. Data are presented as the mean ± SD (*n* = 5). ^#^*p* < 0.05, ^##^*p* < 0.01 compared with H/R group, ^Δ^*p* < 0.05, ^ΔΔ^*p* < 0.01 compare with HCQ 5 group, NS, no statistical significance

To further confirm whether HCQ suppresses NLRP3 inflammasome activation through reduction of CTSB and CTSL activity, we compared the effects of HCQ and Ca074Me on H/R-induced HK-2 cell injury and NLRP3 inflammasome activation. Unlike Ca074Me, HCQ reduced all three cathepsin activity in a dose-dependent manner (Fig. 7a). Then two groups were pretreated with Ca074Me at concentrations of 1 μmol/L (CTSB inhibitor) and 15 μmol/L (CTSB and CTSL inhibitor), respectively. One group was pretreated with HCQ only. Another group was pretreated with HCQ and high concentration of Ca074Me (15 μmol/L). As shown in Fig. 7b, cell viability in Ca074Me 15 μmol/L group was better than Ca074Me 1 μmol/L group, suggesting both CTSB and CTSL are essential in H/R-induced cell injury. Cell viability in HCQ group showed significant improvement compared with Ca074Me 15 μmol/L group, indicating other cathepsin(s) may also be involved in H/R injury in addition to CTSB and CTSL. However, cell viability in HCQ group and HCQ + Ca074Me group showed no statistical significance, suggesting that HCQ attenuates H/R-induced injury primarily by reducing CTSB and CTSL activity. Correspondingly, expression of caspase-1 p10 and IL-1β in the culture supernatants (Figs. 7c, d), and mRNA levels of IL-1β, IL-6, TNF-α, and MCP-1 (Fig. 7e) in HCQ group and HCQ + Ca074Me group also showed no statistical significance, which confirmed that HCQ suppresses NLRP3 inflammasome through downregulation of CTSB and CTSL activity. In addition, we also transfected CTSB and CTSL siRNA into HK-2 cells to further verify our findings. Similarly, protein expression of caspase-1 p10 and IL-1β (Figs. 7f, g) in the culture supernatants, and gene expression of pro-inflammatory cytokines (Fig. 7h) also showed no statistical significance between HCQ group and CTSB + CTSL siRNA + HCQ group. These data suggested that HCQ blocks NLRP3 inflammasome activation by reducing CTSB and CTSL activity.

## Discussion

Interstitial inflammation is a major pathological feature of ischemic AKI, which could be a potential therapeutic target to mitigate the severity of AKI. In this study, we have demonstrated that pretreatment with HCQ could significantly attenuate inflammatory responses in renal I/R injury by inhibiting cathepsin B/L-mediated NLRP3 inflammasome activation.

CQ and its analog HCQ, originally antimalarial drugs, now have been widely used in the treatment of autoimmune diseases because of their anti-inflammatory and immunomodulatory effects^6^. However, their effects on renal inflammation and potential mechanism remains unclear. Yasuda et al. demonstrated that CQ could reduce typical inflammatory cytokines TNF-α and IL-10 production in sepsis-induced AKI through inhibition of toll-like receptor 9^35^. Fang et al. found that CQ protects aganist liver damage in the early phase by inhibiting expression of inflammatory cytokines, such as TNF-α, IL-6 and IL-1β^13^. In the present study, we demonstrated that HCQ has clear anti-inflammatory effects in I/R-induced AKI, which is supported by the finding that HCQ pretreatment markedly reduced the interstitial infiltration of immune cells and expression of inflammatory cytokines.

Emerging evidence has suggested an important role for the NLRP3 inflammasome in the pathogenesis of acute and chronic injury in a wide spectrum of renal diseases including ischemic AKI^21–24,36^. In addition to classical immune cells, activation of the NLRP3 was also demonstrated in renal parenchymal cell types including podocytes and TECs^22,36^. Of note, the NLRP3 inflammasome is a critical mediator of ischemic AKI, such that NLRP3 deficiency protects against renal I/R injury and enhances the reparative response^26,27^. Consistently, our data in murine and HK-2 cells indicated that the activation of NLRP3 inflammasome was increased by I/R or H/R injury, but was significantly remitted by HCQ pretreatment, suggesting that inhibition of NLRP3 inflammasome activation is responsible for the anti-inflammatory effects of HCQ in renal I/R injury. However, the mechanism of I/R-triggered NLRP3 inflammasome activation is still unknown. Although the inflammasome is active in both immune and non-immune cell lineages, our in vitro data with HK-2 cells indicated that HCQ could have a protective effect in AKI directly at the level of the TECs.

Our data had provided a new insight into the mechanism through which HCQ inhibits the activation of NLRP3 inflammasome. First, we found that HCQ pretreatment down-regulated I/R or H/R-induced NF-κB signaling, a known activator of NLRP3^37,38^, suggesting HCQ could inhibit the priming signal of NLRP3 inflammasome. Attenuated NF-κB activity with HCQ therapy would account for the blunted expression of NF-κB-dependent cytokines detected in our study, including IL-1β and TNF-α. Second, as intracellular acidosis during ischemia would favor lysosomal hydrolase (cathepsin) activity^39–41^, we further investigated whether lysosomal cathepsin is capable of activating NLRP3 during I/R injury. The results clearly demonstrated that I/R injury causes the redistribution of lysosomal cathepsin from lysosomes to the cytoplasm due to lysosomal rupture, and CTSB/CTSL (not CTSD) are involved in I/R-induced NLRP3 inflammasome activation. Thus, HCQ appears to inhibit NLRP3 inflammasome activation by down-regulating CTSB and CTSL activity.

HCQ is well-known as autophagy inhibitor by reducing autophagosome clearance. Some increasing evidence suggested that autophagy provides a protective response to various types of pathological injuries including I/R injury. Suppression of autophagy by CQ worsens ischemic kidney injury^42,43^. In our study, additional experiments showed that autophagic flux was blocked by HCQ, however, the inhibition of autophagy was most significant when HCQ presented after I/R injury. This is supported by the observation that HCQ administration after reperfusion or reoxygenation showed much more LC3-II and p62 accumulation than that pretreated before ischemia or hypoxia. (Supplementary Fig. 3). In addition, some authors pointed out autophagy can also act as a foe of I/R injury, depending on the severity of ischemia, the degree of autophagy activation, the phase of I/R injury and probably other still unknown factors^44,45^. Liu et al^46^. recently demonstrated the existence of autophagy-dependent cell death in a pathological model of cerebral ischemia and various cell lines. Kanayama et al^47^. found that autophagy enhances NF-κB signaling in macrophages in kidney by sequestering NF-κB inhibitor A20. The dual role of HCQ in anti-inflammation and anti-autophagy need to be further studied.

**Fig. 8:**
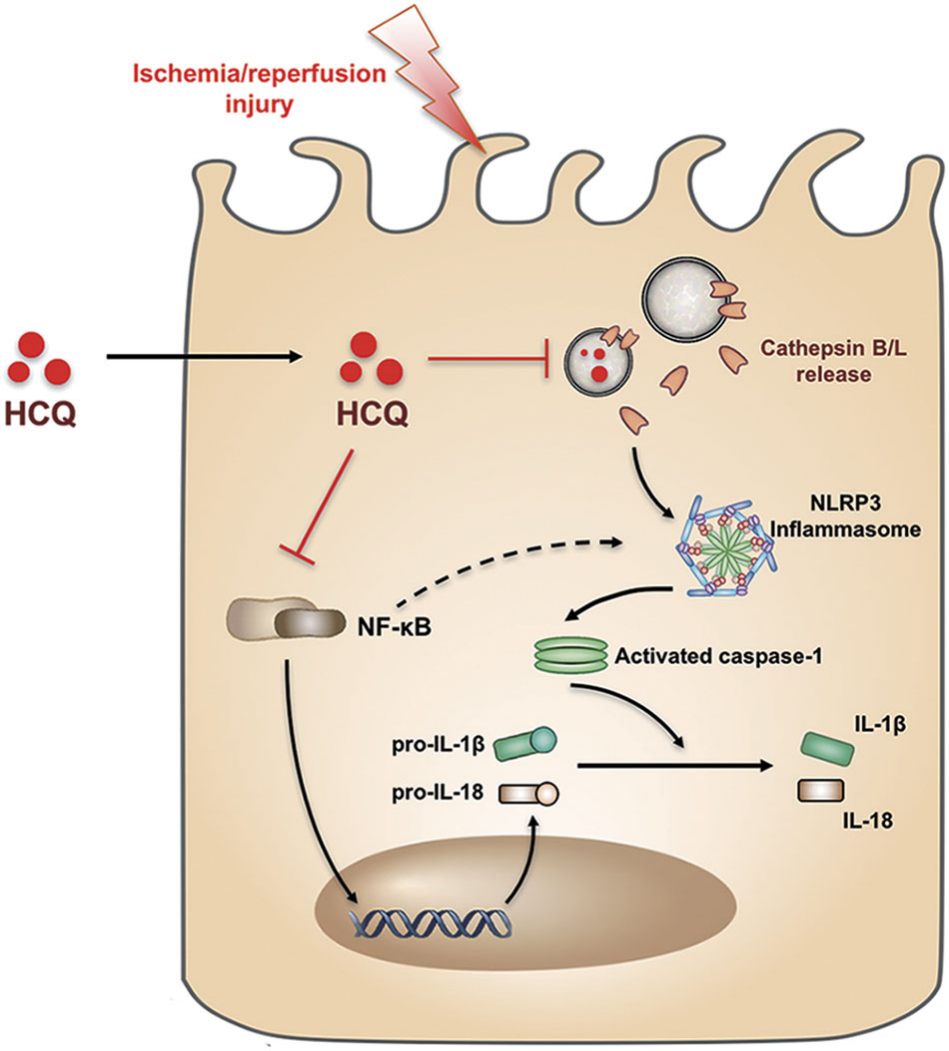
Schematic diagram of this study. HCQ protects renal I/R injury through the down-regulation of NF-κB signaling and inhibition of cathepsin B/L mediated NLRP3 inflammasome activation

Taken together, we have demonstrated that HCQ pretreatment exerts a renoprotective effect in I/R injury. The protective actions of HCQ include inhibition of NF-κB signaling and NLRP3 inflammasome activation, the latter via down-regulation of CTSL/CTSB activity (Fig. 8). These findings warrant translation investigations regarding the use of HCQ for the treatment of ischemic AKI.

## Materials and methods

### Animal models of renal I/R injury

Male C57BL/6J mice (8 week–10 week old), weighing 20–22g were purchased from the National Model Animal Center of Nanjing University and were studied using protocols approved by the Ethics Committee of Southeast University. The animals were fed in a 12/12 h light/dark cycle and free access to food and water. The mice were divided randomly into four groups: the sham-saline group; the sham-HCQ group; the IRI-saline group; and the IRI-HCQ group. Each group has 8 mice. For ischemia, the mice were anesthetized with pentobarbital sodium (50 mg/kg, i.p.) on the day of surgery. Then, both renal pedicles were clamped by microaneurysm clamps for 30 min, as previously reported^48^. For reperfusion, the clamp was released and the kidney was monitored by a color change to confirm blood reflow before suturing the incision. Sham operations were performed with exposure of both kidneys but without renal pedicle clamping. Mice form different groups were sacrificed after reperfusion for 24 h. In HCQ-pretreated groups and the saline-pretreated groups as the solvent contrast, 10 mg/kg/day of HCQ and vehicle solution were respectively administered by gavage for 7 days before the procedure. During the surgery, all animals were placed on a heating blanket to maintain body temperature.

To analyze the effects of CA074ME on I/R injury, mice were dosed with 10 mg/kg CA074ME, administered daily by intraperitoneal injection, for 3 days prior to surgery. Mice in the sham operation were administered with saline. Then animal models of renal I/R injury were carried out as previously described. Mice form different groups were sacrificed after reperfusion for 24 h.

### Renal function and histology

Renal function was monitored by serum creatinine. After reperfusion for 24 h, blood samples were collected and coagulated at room temperature, followed by centrifugation to get serum. Serum creatinine levels were measured with commercial kits (Jiancheng, China) according to the manufacturer’s instructions. For histology, kidneys were fixed with 4% formaldehyde and embedded in paraffin for staining with H&E for morphological analysis. Tissue damage was examined using a tubular damage score as previously described: 0, no damage; 1, <25%; 2, 25~50%; 3, 50~75%; 4, >75%^43^.

### Cell culture

The human proximal tubular epithelial cell line HK-2 was purchased from the American Type Culture Collection (ATCC). Cells were cultured in DMEM-Ham’s-F-12 medium (Gibco, USA) supplemented with 10% fetal bovine serum (Gibco, USA), human recombinant epidermal growth factor (5 ng/ml) (Gibco, USA), and penicillin (100 IU/ml) and streptomycin (100 μg/ml) in a humidified atmosphere of 5% CO_2_ and 95% O_2_ at 37 °C.

### Cell hypoxia/ reoxygenation

The HK-2 cells were plated in 35 mm dishes at a density of 10^6^ cells/ dish and incubated until they reached approximately 90% confluence for experiment. The cells in hypoxia/ reoxygenation (H/R) group were cultured for 12 h under hypoxic conditions (1% O_2_, 94% N_2_, and 5% CO_2_) in medium without nutrients (glucose-free, serum-free) to induce hypoxic injury. After hypoxic treatment, the cells were transferred back to regular culture medium with oxygen for 2 h for reoxygenation. Control cells were incubated in complete culture medium in a regular incubator (5% CO_2_ and 95% air). The cells in HCQ-treated groups were pretreated with HCQ (0.5, 2.5, and 5 μmol/L) for 12 h before H/R operation.

### HCQ toxicity assay and cell viability assay

The HK-2 cells were seeded into 96-well plates at a density of 10^4^ cells/ well and reached 70% confluence by next day for experiment. Then, the cells were pretreated with various concentrations of HCQ (TCI, Japan) for 24 h at 37 °C. And the toxicity of HCQ in the cells was assayed by the CCK-8 method (Vazyme, China) according to the manufacturer’s instructions. To analyze the effect of HCQ on cell viability in HK-2 affected by H/R, the cells were pretreated with HCQ for 12 h, and the H/R protocol was performed. Cell viability was assayed according to the CCK-8 method.

### Immunohistochemistry and immunofluorescence staining

Paraffin-embedded sections from the kidney cortex were used for immunohistochemistry. Briefly, the sections were incubated with primary antibodies to F4/80, neutrophil (Abcam, USA), NLPR3 (Adipogen, USA), ASC, caspase-1 (Santa Cruz Biotechnology, USA) overnight at 4 °C. The sections were then analyzed using streptavidin peroxidase detection system (Maixin, China) according to the manufacturer’s protocol. The reaction was developed using DAB substrate kit (Maixin, China), and counterstaining was performed using hematoxylin.

For analysis and localizing the expression of the NLRP3 and cathepsin B, immunofluorescence staining of tissue sections or formaldehyde-fixed cells was performed using anti-NLRP3, anti-cathepsin B antibodies (Abcam, USA), respectively in a humidified chamber overnight at 4 °C, followed by incubation with an Alexa fluorescein-labeled secondary antibodies (Invitrogen, USA) for 1 h. Cell nuclei were stained with DAPI. Immunostained samples were visualized under a confocal microscope. Immunofluorescence for p65 (Cell Signaling Technology, USA) was similarly performed. Quantification of intensity of p65 in nuclear was performed by measuring area, integrated density, and mean gray value using Image-Pro Plus.

### Supernatant ELISA detection

The IL-1β and TNF-α contents in cell-free supernatants were measured using ELISA kits (R&D Systems, USA), according to the manufacturer’s instructions.

### Cathepsin activity assay

Cathepsin B, cathepsin D, and cathepsin L activity was measured using cathepsin B, D, and L activity assay kit (Abcam, USA), according to the manufacturer’s instructions. The fluorescence was measure by VersaFluor Fluorometer (Bio-Rad, Hercules, CA) respectively.

### Cathepsin siRNA transfection

Transfection was performed when the HK-2 cells were cultured to 70% confluence according to the manufacturer’s protocol (Invitrogen, USA). Briefly, the cathepsin B-targeted, cathepsin D-targeted, cathepsin L-targeted siRNA, control siRNA (GenePharma, China) and Lipofectamine RNAimax (Invitrogen, USA) were mixed with Opti-MEM (Gibco, USA) respectively at room temperature for 5 min before seeding in a well containing serum-free DMEM-Ham’s-F-12 medium.

### Quantitative real-time PCR assay

The total RNA from HK-2 cells was extracted using the RNAiso plus reagent, and cDNA was then synthesized using a reverse transcription system kit (Takara, Japan) according to the manufacutrer’s instructions. Real-time RT-PCR was performed using an ABI PRISM 7300 real-time PCR System (Applied Biosystems, USA). This assay was used to determine the levels of IL-1β, MCP-1, TNF-α, IL-6. The results were analyzed using the comparative cycle threshold (ΔΔCt) method. Primer sequences were listed in the Supplementary Table 1.

### Western blotting assay

The protein lysates from the HK-2 cell and kidney tissues were prepared following standard protocols according to the manufacturer’s protocol, and the protein content was determined using the BCA protein assay kit (KeyGEN, China). The protein of culture supernatants was extracted using StrataClean Resin (Agilent Technologies, USA). And then the proteins samples were separated by Bis-Tris Gel (Invitrogen, USA) and transferred onto PVDF membranes (Millipore, USA) using a wet-transfer system. Membranes were blocked in 5% BSA in TBS-T for 1 h at room temperature and were incubated with primary antibodies overnight at 4 °C. Then membranes were washed and incubated with secondary horseradish peroxidase-conjugated antibodies for 2 h at room temperature, and the signals were detected using an ECL advanced system (GE Healthcare, UK). Intensity values expressed as the relative protein expression were normalized to β-actin and GAPDH.

Primary antibodies used were anti-NLRP3 (AG-20B-0014-C100, Adipogen); anti-ASC (SC-22514-R, Santa Cruz Biotechnology); anti-mouse Caspase-1 (sc-514, Santa Cruz Biotechnology); anti-human Caspase-1 (sc-515, Santa Cruz Biotechnology); anti-IL-1β (ab-9722, Abcam); anti-KIM-1 (ab-47634, Abcam); anti-NF-κB p65 (8242, Cell Signaling Technology); anti-NF-κB p-p65 (3033, Cell Signaling Technology); anti-GAPDH (ab-181602, Abcam); anti-β-actin (ab-8226, Abcam). Secondary HRP-conjugated antibodies used were anti-mouse IgG and anti-rabbit IgG (Vazyme).

### Statistical analysis

All data are expressed as the mean ± standard deviation (SD), and results were analyzed using one-way analysis of variance in SPSS 20.0 statistical software. Differences with a *p* value less than 0.05 were considered statistically significant.

## Electronic supplementary material

Supplementary Figure and Table Legends

Supplementary Figure 1

Supplementary Figure 2

Supplementary Figure 3

Supplementary Figure 4
