# Supplementary material for: Hydroxychloroquine attenuates renal ischemia/reperfusion injury by inhibiting cathepsin mediated NLRP3 inflammasome activation
Source: Cell Death Dis. 2018 Mar 2;9(3):351. doi: 10.1038/s41419-018-0378-3 (PMC5834539; doi:10.1038/s41419-018-0378-3)
Supplement: Supplementary file 1 — Supplementary Figure and Table Legends [file 41419_2018_378_MOESM1_ESM.docx]

**Supplementary Figure and Table Legends**

**Supplementary Figure 1** HCQ does not affect the expression of NLRP3 components and pro-inflammatory cytokines in HK-2 cells under normal conditions. HK-2 cells were treated with different doses of HCQ for 12 h. (A) Western blots of NLRP3 components in cell lysates from HK-2 cells treated with HCQ. (B) mRNA expression levels of IL-1β, IL-6, TNF-α and MCP-1 in HK-2 cells treated with HCQ. Data are presented as the mean ± SD (n=3). There was no significant statistical significance between each group.

**Supplementary Figure 2** Cathepsin siRNA transfection does not affect the expression of NLRP3 components and pro-inflammatory cytokines in HK-2 cells. HK-2 cells were transfected with Ctrl siRNA or Cathepsin (CTSB, CTSD, CTSL) siRNA and 24 h post-transfection cells were collected. (A) Western blots of NLRP3 components in cell lysates from HK-2 cells transfected with siRNA. (B) mRNA expression levels of IL-1β, IL-6, TNF-α and MCP-1 in HK-2 cells transfected with siRNA. Data are presented as the mean ± SD (n=3). There was no significant statistical significance between each group.

**Supplementary Figure 3** Effects of HCQ on autophagy flux in vivo and in vitro. In vivo, animal models of renal I/R injury were carried out as previously described. HCQ was administered at dosage of 10 mg/kg/day by gavage for 3 days before ischemia (IRI-HCQ pretreatment group) or administered for 3 days after reperfusion (IRI-HCQ treatment group). Mice were sacrificed after reperfusion for 3 days. In vitro, cell hypoxia/reoxygenation were carried out as previously described. HCQ was administered with dosage of 5 μmol/L for 12 h before hypoxia (H/R-HCQ pretreatment group) or administered for 12 h after reoxygenation (H/R-HCQ treatment group) and 12 h post-reoxygenation cells were collected. (A) Western blots of LC3 and p62 in kidney lysates. Primary antibodies used were anti-LC3B (3868, Cell Signaling Technology) and anti-p62 (5114, Cell Signaling Technology). (B) Immunofluorescence staining of LC3 and DAPI. (Bars=50 μm) Data are presented as the mean ± SD (n=6). ## p<0.01, ** p<0.01. (C) Western blots of LC3 and p62 in HK-2 lysates. Primary antibodies used were anti-LC3B (3868, Cell Signaling Technology) and anti-p62 (5114, Cell Signaling Technology). (D) Live cell imaging by confocal microscopy with Cyto-ID staining of autophagy and Hoechst 33342 of nuclear. (Bars=30 μm) Data are presented as the mean ± SD (n=3). # p<0.05, ## p<0.01, * p<0.05, ** p<0.01.

**Supplementary Figure 4** Negative controls for immunofluorescence staining. (A) Negative control related to Fig. 3F. (Image magnification: ×1000) (B) Negative control related to Fig. 4E. (Image magnification: ×1000) (C) Negative control related to Fig. 5I. (Image magnification: ×1000)

**Supplementary Table 1** Primers used in this study

| Gene | Forward | Reverse |
| --- | --- | --- |
| β-actin-HOMO | CTACCTCATGAAGATCCTCACCGA | TTCTCCTTAATGTCACGCACGATT |
| MCP-1-HOMO | CTTGGGTTGTGGAGTGAGTGT | AGCAGAAGTGGGTTCAGGATT |
| TNF-α-HOMO | CGAAGTGGTGGTCTTGTTGCT | CCCGACTATCTCGACTTTGCC |
| IL-1β-HOMO | GTGGTGGTCGGAGATTCGTAG | GAAATGATGGCTTATTACAGTGGC |
| IL-6-HOMO | GCTCTGGCTTGTTCCTCACTA | AATCATCACTGGTCTTTTGGAG |
| CTSB-HOMO | TGTTCTTGCGACTCTTGG | GAAGGTTGACGAGGATGAC |
| CTSD-HOMO | GCCAGCACAGAAACAGAG | CACAGAACAAAACAGCAAGT |
| CTSL-HOMO | TATTGGCTGGTGAAGAACA | GGCTGGTAGACTGAAGATGA |
| β-actin-MUS | GGGAAATCGTGCGTGAC | AGGCTGGAAAAGAGCCT |
| MCP-1-MUS | TTGAGGTGGTTGTGGAAAAGG | GTGCTGACCCCAAGAAGGAAT |
| TNF-α-MUS | AGACAGAGGCAACCTGACCAC | GCACCACCATCAAGGACTCAA |
| IL-1β-MUS | GGTAAGTGGTTGCCCATCAGA | GTCGCTCAGGGTCACAAGAAA |
| IL-6-MUS | GTCACCAGCATCAGTCCCAAG | CCCACCAAGAACGATAGTCAA |
