# Supplementary figures and images for: Hydroxychloroquine attenuates renal ischemia/reperfusion injury by inhibiting cathepsin mediated NLRP3 inflammasome activation
Source: Cell Death Dis. 2018 Mar 2;9(3):351. doi: 10.1038/s41419-018-0378-3 (PMC5834539; doi:10.1038/s41419-018-0378-3)

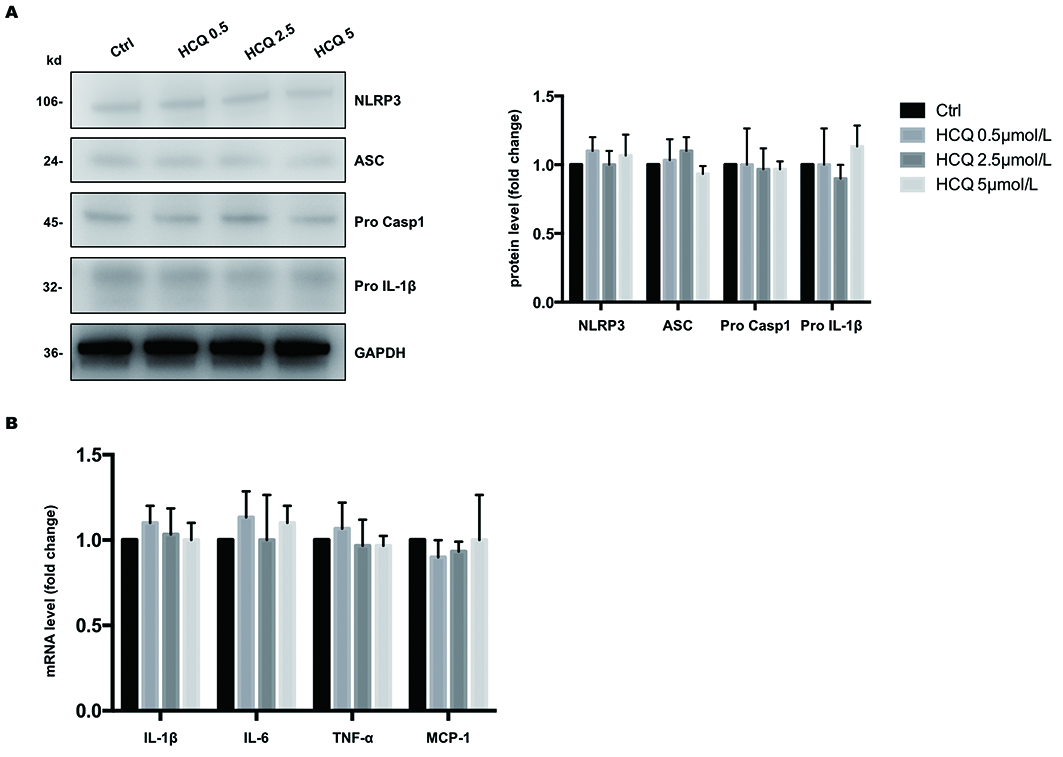

Supplement: Supplementary file 2 — Supplementary Figure 1 [file 41419_2018_378_MOESM2_ESM.tif]

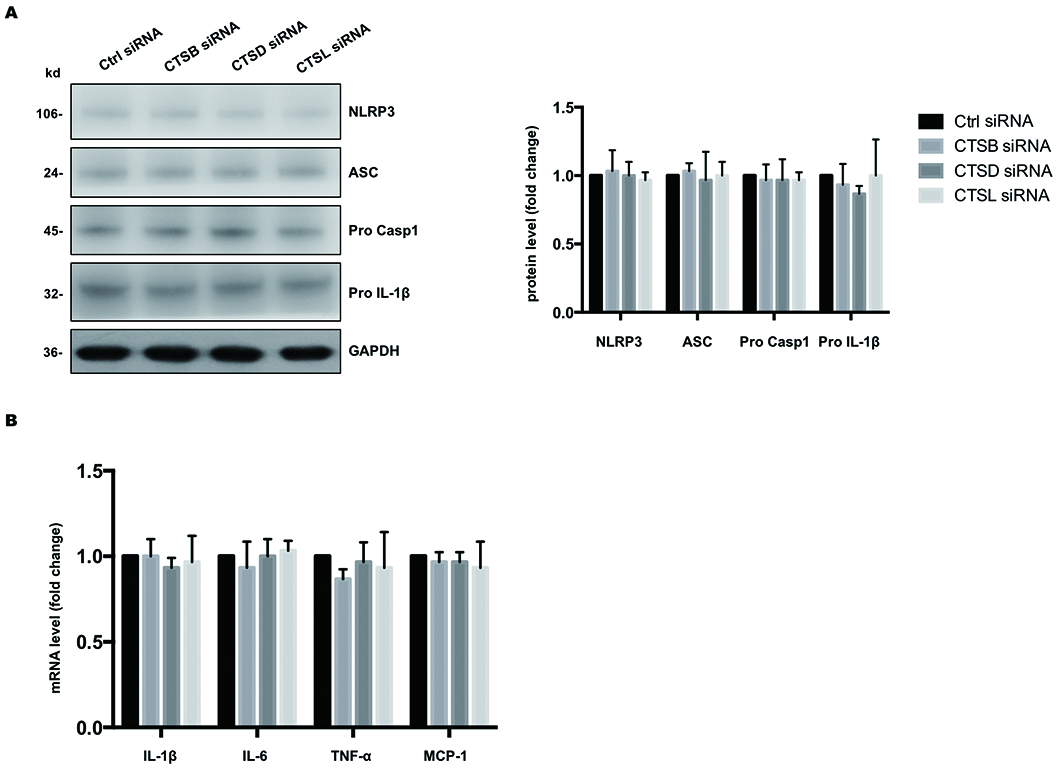

Supplement: Supplementary file 3 — Supplementary Figure 2 [file 41419_2018_378_MOESM3_ESM.tif]

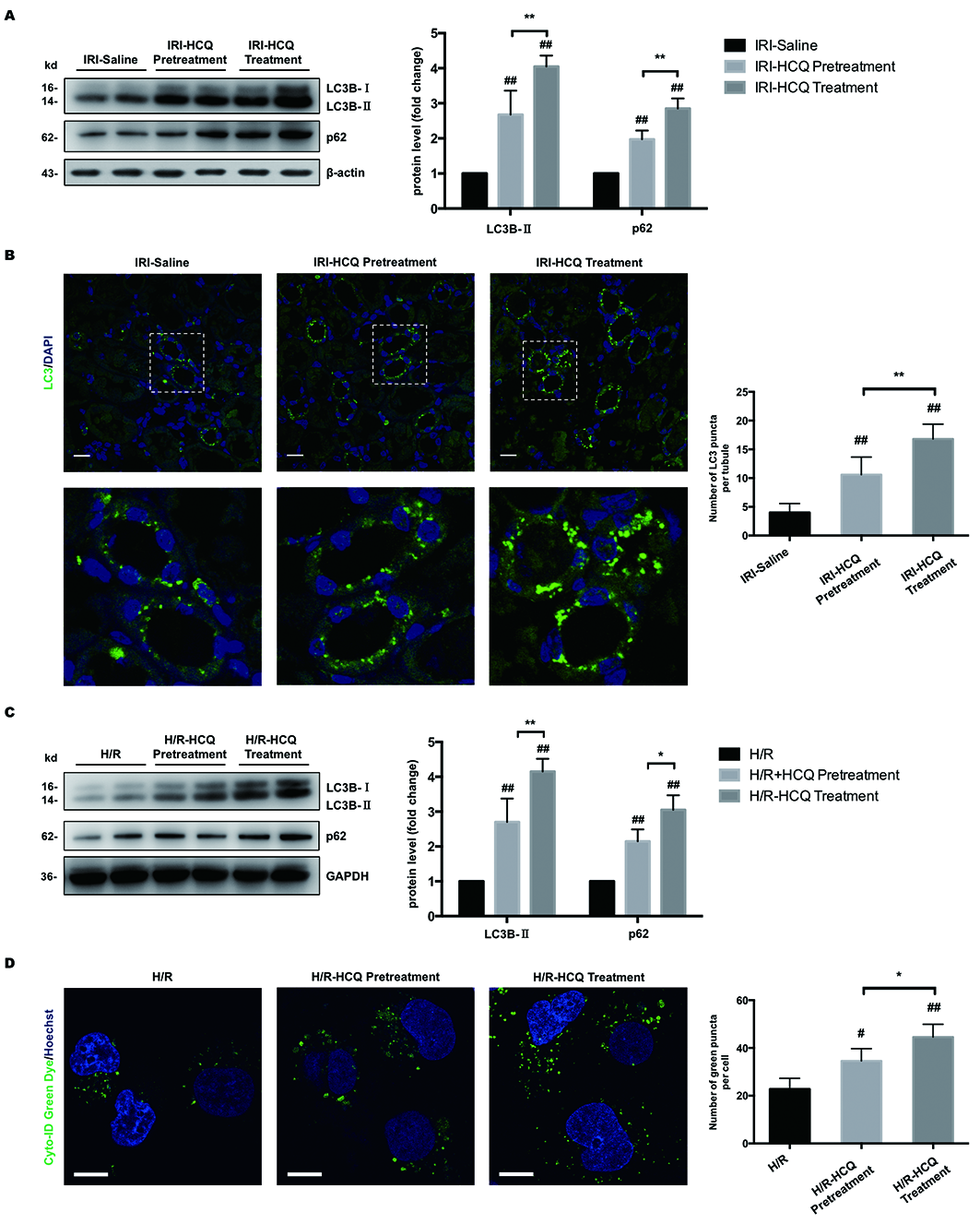

Supplement: Supplementary file 4 — Supplementary Figure 3 [file 41419_2018_378_MOESM4_ESM.tif]

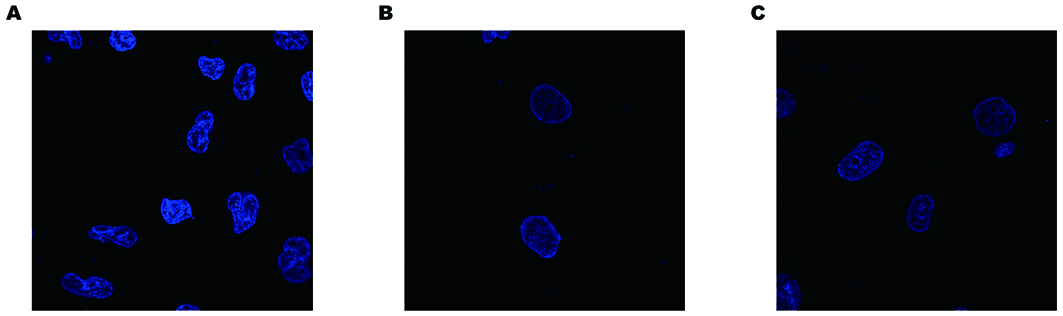

Supplement: Supplementary file 5 — Supplementary Figure 4 [file 41419_2018_378_MOESM5_ESM.tif]
